# Targeting metabotropic glutamate receptors for symptomatic and disease-modifying treatment in Parkinson’s disease

**DOI:** 10.1038/s41531-025-01138-1

**Published:** 2025-10-10

**Authors:** Marika Alborghetti, Alessia Ceccherelli, Matteo Caridi, Ferdinando Nicoletti, Giuseppe Battaglia, Valeria Bruno

**Affiliations:** 1 Department of Neuroscience, Mental Health and Sensory Organs, Sapienza University, Rome, Italy; 2 Department of Neurology, IRCCS Neuromed, Pozzilli, Italy; 3 Department of Physiology and Pharmacology, Sapienza University, Rome, Italy; 4 Hematology Unit-ASL Viterbo, Santa Rosa Hospital, Viterbo, Italy; 5 Department of Molecular Pathology, IRCCS Neuromed, Pozzilli, Italy

**Keywords:** Diseases, Drug discovery, Neurology, Neuroscience

## Abstract

Degeneration of substantia nigra pars compacta dopaminergic neurons is the “hallmark” of Parkinson’s disease (PD) and is responsible for motor signs. Other neurotransmitter systems are responsible for non-motor symptoms that may precede by decades the clinical onset of motor symptoms. The pathophysiology is complex and neurodegeneration involves excitotoxicity mechanisms and neuroinflammation. L-DOPA is the “gold” symptomatic therapy but does not halt the progression of the disease. Therefore, neuroprotective strategies are highly demanded. Metabotropic glutamate (mGlu) receptors have emerged as potential pharmacological targets because they modulate glutamatergic, GABAergic, and dopaminergic neurotransmissions, and have been implicated in mechanisms of neurodegeneration and neuroinflammation. Thus, mGlu receptors represent valuable targets for the development of new disease-modifying and symptomatic therapies for PD. This review highlights the role of individual mGlu receptor subtypes in the pathophysiology of motor and non-motor symptoms of PD and in mechanisms that contribute to the progression of the disease.

## Searching for broad-spectrum drugs targeting mechanisms that underlie motor and non-motor signs, neuroinflammation and neurotoxicity in Parkinson’s disease

L-DOPA combined with peripheral inhibitors of L-amino acid aromatic decarboxylase (LAAD) is the “gold” standard in the treatment of Parkinson’s disease (PD), but, in the long-term, L-DOPA treatment is complicated by the onset of motor and non-motor fluctuations and involuntary movements (L-DOPA-induced dyskinesias or LIDs). The efficacy of other drugs, e.g., dopamine receptor agonists, MAO_B_ inhibitors and anticholinergic agents, is suboptimal, and there are no drugs that slow the progression of PD acting as disease-modifying agents^1^. Phase 2 studies with monoclonal antibodies directed against aggregates of α-synuclein (e.g., cimpanemab and prasinezumab) showed no significant changes in the Movement Disorder Society-sponsored Revision of the Unified Parkinson’s Disease Rating Scale (MDS-UPDRS) score, although prasinezumab caused a near-to-significant improvement in motor symptoms (MDS-UPDRS III), and clinical studies with prasinezumab are still ongoing (PADOVA trial NCT04777331). Other drugs, including monoclonal antibodies, small molecule inhibitors/activators, antisense oligonucleotides, and siRNAs, are under development as disease-modifying agents in PD^2,3^. A poor brain penetration and high costs are potential pitfalls in the clinical development of these new therapeutic agents. Thus, there are several unmet needs in the treatment of PD, and the development of drugs targeting mechanisms that lie at the core of the disorder is urgently needed. New broad-spectrum agents should be effective on motor and non-motor symptoms, and target some of the mechanisms underlying the progression of PD.

PD is a multifactorial disorder characterized by a progressive degeneration of dopaminergic neurons in the substantia nigra pars compacta (SNpc) and neuronal degeneration in other brainstem nuclei, such as the locus coeruleus (LC), the raphe nuclei, and the dorsal nucleus of vagus nerve. Dopamine denervation in the dorsal striatum causes a hyperactivity of the indirect pathway, which is physiologically inhibited by D2 dopamine receptors, and a reduced activity of the direct pathway, which is physiologically activated by D1 receptors. The resulting abnormality in the basal ganglia motor circuit causes the classical motor signs in PD. Formation of a long-lasting enhancement of excitatory synaptic transmission at the synapse between cortico-striatal glutamatergic fibers and D1-expressing neurons of the direct pathway is a key mechanism in the pathogenesis of LIDs. Thus, new drugs developed for an optimal and long-lasting control of motor symptoms in PD should target regulatory mechanisms that restrains the hyperactivity of the indirect pathway and/or support the activity of the direct pathway without enhancing the occurrence of LIDs if given in combination with L-DOPA.

It is currently believed that neuronal death in PD is caused by the formation of pathological aggregates of α-synuclein (found in Lewy bodies), which can spread to neighbor neurons through a mechanism of seeding and template^4,5^. However, Lewy bodies are not found in monogenic PD caused by mutations of parkin^6^, a protein playing a key role in the mitochondrial quality control (MQC)^7^. Aggregates of α-synuclein may inactivate parkin, leading to mitochondrial dysfunction^8^. The MQC impairment (i.e., the impairment of mitophagy, mitochondrial fusion, fission, and biogenesis, and formation of mitochondrial vesicles) may have devastating consequences for SNpc neurons, which are not protected against oxidative stress. Damaged mitochondria generate reactive oxygen species (ROS) through a defective respiratory chain, and, in addition, the opening of mitochondrial pores may trigger different types of cell death, such as apoptosis, necrosis, ferroptosis and pyroptosis. Interestingly, the exit of formylated mitochondrial proteins, cardiolipin, and mitochondrial DNA and RNA may trigger innate immunity in SNpc and other vulnerable neurons, contributing to the cascade of events causing neuroinflammation since the early phase of neurodegeneration^9,10^.

Microglia is a key player in neuroinflammation associated with PD and other neurodegenerative disorders^11^. A sustained low-grade neuroinflammation mediated by cells of innate immunity, such as activated microglia and macrophages, is increasingly recognized as a driver of PD progression^12^.

Activated microglia produces pro-inflammatory cytokines, chemokines and complement proteins, contributing to the overall formation of ROS in PD^11,12^. Microglia-derived interferon-γ may damage SNpc neurons, as shown by the use of an in vitro model in which mixed microglia/midbrain neurons were challenged with rotenone^13^. In macaques treated with the parkinsonian toxin, 1-methyl-4-phenyl-1,2,3,6-tetrahydropyridine (MPTP), microglial cells are polarized toward dopaminergic neurons with engulfing gliaptic contacts, even years after the neurotoxic insult^14^. Interestingly, there is a reciprocal cause-to-effect relationship between α-synuclein aggregates and microglia activation. Aggregated α-synuclein can amplify microglia activation induced by pro-inflammatory cytokines, and can also activate the NLRP-3 inflammasome in microglia^10,15–18^. On the other hand, microglia-derived neuroinflammation may enhance tyrosine nitrosylation of α-synuclein, with ensuing protein aggregation^19^. Nitrosylated α-synuclein is detected in cervical lymph nodes from MPTP-lesioned mice and can by-pass immunological tolerance, thereby inducing an adaptive immune response^20^. In patients affected by PD, T cells with specific MHC alleles can induce α-synuclein-related autoimmunity^21^, with the Y39 epitope being close to α-synuclein mutations causing monogenic PD^22^. Aggregation of α-synuclein resulting from Tyr-nitrosylation or other mechanisms causes a loss of immune tolerance, leading to CD4^+^ and CD8^+^ T cell infiltrates^23–25^. T cell infiltration is also observed in the mouse striatum and SNpc after acute injection of MPTP^26^. Thus, neuroinflammation is a potential target for novel disease-modifying drugs in PD.

Excitotoxicity resulting from overactivation of N-methyl-D-aspartate (NMDA) receptors and other types of glutamate receptors is another established component of neurodegeneration in PD^27–29^. In addition, a glutamatergic hyperactivity contributes to the pathophysiology of motor and non-motor symptoms in PD and to the induction and expression of maladaptive synaptic plasticity underlying LIDs^30,31^. Hence, drugs that modulate glutamatergic neurotransmission cater the potential to act as both symptomatic and disease-modifying agents in PD.

Drugs targeting ionotropic glutamate receptors (e.g., AMPA, NMDA or kainate receptors) have limited value as novel broad-spectrum drugs in PD for their strong impact on excitatory synaptic transmission in the CNS. In contrast, metabotropic glutamate (mGlu) receptors modulate synaptic transmission and display pleiotropic functions in neurons, astrocytes and microglia.

mGlu receptors form a family of eight subtypes (mGlu1 to -8), which are found, with the exception of mGlu6, in brain regions involved in the pathophysiology of motor and non-motor signs of PD^32,33^.

**Fig. 1:**
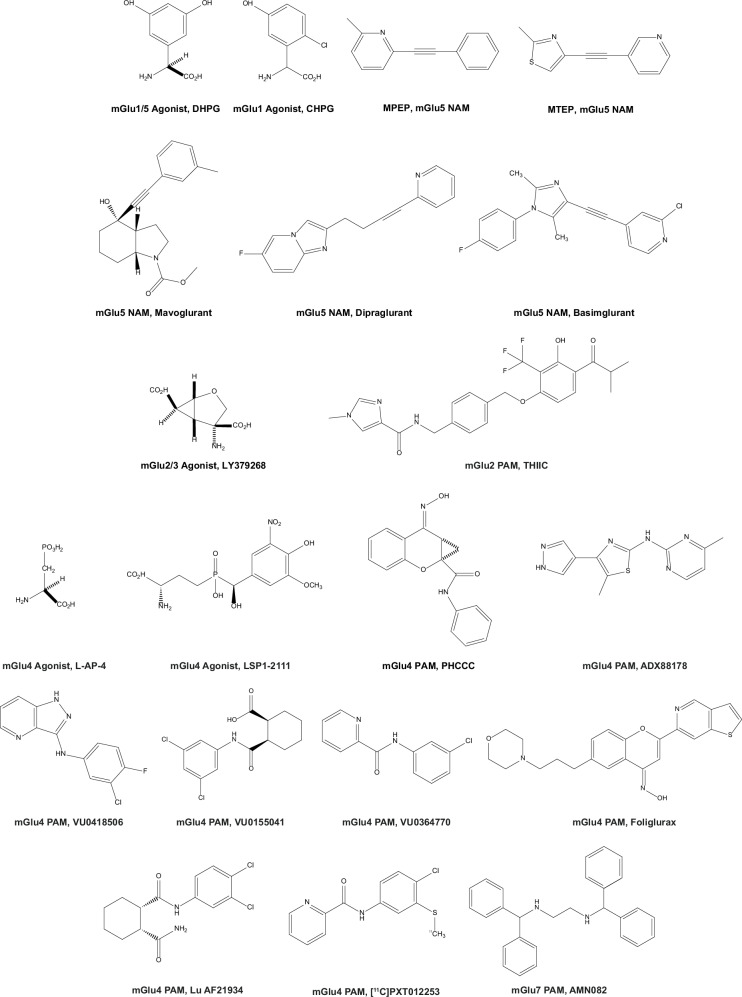
Selected ligands of mGlu receptor subtypes. Mavoglurant, dipraglurant, and foliglurax have been clinically developed for the treatment of PD; basimglurant has been clinically developed for the treatment of major depressive disorder (see text). NAM negative allosteric modulator, PAM positive allosteric modulator. Chemical names are detailed in the text.

Here, we discuss the role of individual mGlu receptor subtypes in the pathophysiology of motor and non-motor symptoms of PD, and their involvement in mechanisms of neuroinflammation and excitotoxicity, highlighting their potential value as therapeutic drug targets in PD. A list of mGlu receptor ligands used in preclinical models of Parkinsonism and in clinical trials is shown in Fig. 1 (see also Tables 1 and 2).

**Table 1:** Preclinical studies on mGlu receptors

First author (Reference number)	Receptor subtype	Model	Intervention	Main outcome(s)
Battaglia et al.^74^	mGlu5	MPTP-treated mice	MPEP and SIB1893	↓ nigrostriatal damage
Masilamoni et al.^75^	mGlu5	MPTP-treated monkeys	MTEP	↓ dopaminergic and noradrenergic neuronal loss
Ambrosi et al.^81^	mGlu5	6-OH-DA lesioned rats	MPEP	↓ akinesia
Lin et al.^89^	mGlu5	hemi-parkinsonian rat model of dyskinesias	MTEP	↓ mRNA levels of prodynorphin and proenkephalin
Mela et al.^90^	mGlu5	6-OH-DA lesioned rats treated with L-DOPA	MTEP	↓ abnormal involuntary movements
Morin et al.^92^	mGlu5	MPTP-treated monkeys cured or not with L-DOPA	MPEP and MTEP	antidyskinetic effect
Morin et al.^93^	mGlu5	MPTP-treated monkeys cured with L-DOPA	MPEP	chronic antidyskinetic effect
Ko et al.^96^	mGlu5	MPTP-treated monkeys cured with L-DOPA	Fenobam and Amantadine	antidyskinetic effect
Dupre et al.^100^	5-HT(1A)	6-OH-DA lesioned rats treated with SKF81297	8-OH-DPAT	↓ abnormal involuntary movements
Iderberg et al.^102^	mGlu5 and 5-HT1A/1B	6-OH-DA lesioned rats treated with L-DOPA or SKF81297	MTEP and/or 8-OH-DPAT/CP94253	↓ abnormal involuntary movements
Bezard et al.^98^	mGlu5	MPTP-injected macaque	Dipraglurant	↓ dyskinesias
Murray et al.^125^	mGlu2/3	Reserpine-treated rats and 6-OH-DA lesioned rats	LY379268	↑ locomotor activity in reserpine-treated rats, ↑ TH neurons in 6-OH-DA lesioned rats
Di Menna et al.^57^	mGlu3	mGlu3^–/–^ mice treated with MPTP	–	↑ nigrostriatal damage and microglial activation
Marino et al.^65^	mGlu4	Reserpine-treated rats	PHCCC	↓ akinesia
Le Poul et al.^133^	mGlu4	Haloperidol-treated rats and 6-OH-DA lesioned rats	ADX88178	↓ haloperidol-induced catalepsy ↓ forelimb akinesia (in association with low dose L-DOPA)
Jones et al.^134^	mGlu4	Haloperidol-treated rats and 6-OH-DA lesioned rats	VU0364770	↓ haloperidol-induced catalepsy ↓ forelimb asymmetry and attentional deficits in 6-OH-DA lesioned rats
Bennouar et al.^135^	mGlu4	Haloperidol-treated rats and 6-OH-DA lesioned rats	Lu AF21934	↓ haloperidol-induced catalepsy ↓ akinesia (in association with low dose L-DOPA)
Charvin et al.^143^	mGlu4	MPTP-injected macaque treated with or without L-DOPA	Foliglurax	↓ motor symptoms, ↓ dyskinesias
Beurrier et al.^63^	mGlu4	Haloperidol-treated rats	LSP1-2111	↓ haloperidol-induced catalepsy
Iderberg et al.^141^	mGlu4	6-OH-DA lesioned rats treated with L-DOPA	VU0364770 or LSP1-2111	no antidyskinetic effects
Broadstock et al.^154^	mGlu4, mGlu7, mGlu8	Reserpine-treated rats	PHCCC, AMN082, (S)-3,4-DCPG	↓ akinesia
Greco et al.^155^	mGlu7	Haloperidol-treated rats and 6-OH-DA lesioned rats	AMN082	↓ haloperidol-induced catalepsy, ↓ apomorphine-induced rotations in 6-OH-DA lesioned rats
Konieczny et al.^156^	mGlu7	Reserpine-treated rats and haloperidol-treated rats	AMN082	↓ haloperidol-induced catalepsy
Johnson et al.^158^	mGlu8	Reserpine-treated rats, haloperidol-treated rats and 6-OH-DA lesioned rats	(S)-3,4-DCPG	↓ haloperidol-induced catalepsy ↓ reserpine-induced akinesia ↓ forelimb asymmetry in 6-OH-DA lesioned rats
Battaglia et al.^147^	mGlu4	MPTP-treated mice	PHCCC	↑ striatal levels of dopamine and TH neurons
Austin et al.^149^	mGlu4, mGlu7, mGlu8	Reserpine-treated rats and 6-OH-DA lesioned rats	L-SOP and L-AP4	↓ reserpine-induced akinesia ↑ striatal levels of dopamine and TH neurons in 6-OH-DA lesioned rats
Betts et al.^150^	mGlu4	6-OH-DA lesioned rats	VU0155041	↑ TH neurons, ↓ microglial activation, ↑ motor function

Comprehensive summary of preclinical studies investigating mGlu receptor subtypes in experimental models of Parkinson’s disease. The table includes animal models, receptor targets, pharmacological or genetic interventions, and key outcomes.

**Table 2:** Clinical studies that evaluated activation or inhibition of mGlu receptor subtypes in the management of PD and motor complications

Compound reference	Indications	Study design and outcomes	Results
AFQ506/Mavoglurant Berg et al.^103^	Dyskinesias	Two randomized, double-blind, placebo-controlled, parallel-group, in patients with moderate to severe levodopa-induced dyskinesia (study 1) and severe levodopa-induced dyskinesia (study 2). Primary outcomes were the Lang-Fahn Activities of Daily Living Dyskinesia Scale (study 1), the modified Abnormal Involuntary Movement Scale (study 2), and the Unified Parkinson’s Disease Rating Scale-part III (both studies).	Clinically relevant antidyskinetic effect without changing the antiparkinsonian effects of dopaminergic therapy.
AFQ506/Mavoglurant Stocchi et al.^104^	Dyskinesias	13-week, double-blind, placebo-controlled study to determine the best treatment dosage. The primary outcome was the modified Abnormal Involuntary Movements Scale. Secondary outcomes included the motor evaluation and the severity of motor complications.	A dose-response relationship on the modified Abnormal Involuntary Movements Scale, with 200 mg daily demonstrating the most robust effect. Improvements in dyskinesia were supported by change on Unified Parkinson’s Disease Rating Scale part IV item.
AFQ506/Mavoglurant Trenkwalder et al.^105^	Dyskinesias	Two phase 2 randomized, double-blind studies to evaluate efficacy and safety of immediate-release (study 1) and modified-release (study 2) mavoglurant formulations. The primary outcome was antidyskinetic efficacy, as measured by change in modified Abnormal Involuntary Movement Scale total score.	No improvement of dyskinesia. Adverse events incidence was higher respect to placebo.
Dipraglurant Tison et al.^110^	Dyskinesias	A phase 2a double-blind, placebo-controlled, randomized, 4-week, parallel-group, multicenter dose-escalation clinical trial. The primary outcomes were safety and tolerability. Secondary outcomes included the modified Abnormal Involuntary Movement Scale, UPDRS, and diaries.	Drug was safe and well tolerated. Reduced peak dose dyskinesia on day 1 and on day 14, and across a 3-h post-dose period on day 14.
Foliglurax Rascol et al.^146^	Symptomatic treatment	28-day, multicenter, randomized, placebo-controlled, double-blind clinical trial. The objectives were to evaluate the efficacy and safety of foliglurax in reducing off time and dyskinesias.	Dose-dependent decrease in daily awake off-time. Change from baseline to day 28 in off-time and dyskinesia did not improve.

## Expression and function of mGlu receptors in the basal ganglia motor circuit

mGlu receptor subtypes are divided into 3 groups on the basis of their amino acid sequence, pharmacological profile, and intracellular signaling mechanisms. Group I mGlu receptor subtypes (mGlu1 and mGlu5) are coupled to G_q/11_ proteins and their activation leads to phospholipase-Cβ-mediated hydrolysis of phosphatidylinositol-4,5-bisphosphate, with ensuing formation of inositol-1,4-5-trisphosphate (InsP_3_) and diacylglycerol (DAG). InsP_3_ releases Ca^2+^ from intracellular stores, whereas DAG activates protein kinase C. Both subtypes are predominantly found in the extrasynaptic regions of dendritic spines, where mGlu5 receptors are linked to NMDA receptors and InsP_3_ receptors through a chain of scaffolding and anchoring proteins, such as PSD-95, Shank, and Homer proteins^33,34^. There is a reciprocal functional interaction between mGlu5 and NMDA receptors. Activation of mGlu5 receptors enhances NMDA receptor function by relieving the Mg^2+^ blockade of the NMDA-gated ion channel through a phosphorylation mechanism^35–40^, whereas activation of NMDA receptors amplifies mGlu5 receptor activity by restraining mGlu5 receptor desensitization^41^. mGlu5 receptors are highly expressed by reactive astrocytes, where their activation generates intracellular Ca^2+^ oscillations^42^, and enhances the release of pro-inflammatory cytokines^43^.

**Fig. 2:**
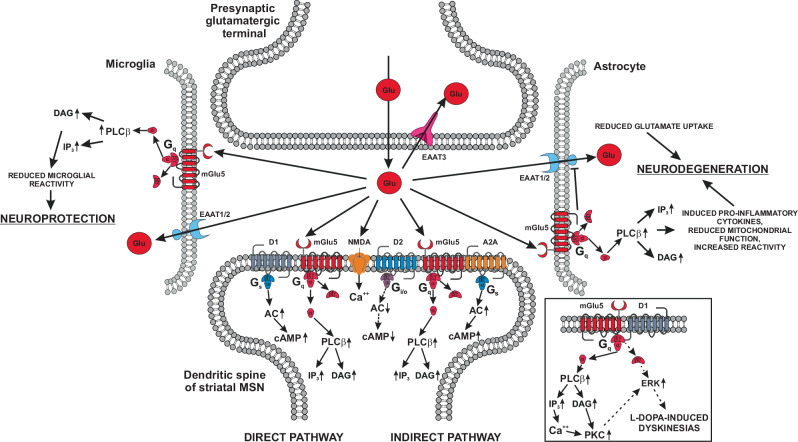
A schematic representation of the potential role of mGlu5 receptors in the pathophysiology and treatment of PD. In dendritic spines of striatal medium spiny neurons (MSN) of the indirect pathway, mGlu5, NMDA, and A2A receptors counteract the inhibitory activity of D2 receptors. Glial (EAAT1/2) and neuronal (EAAT3) glutamate transporters are also shown. Formation of the heteromeric complex between mGlu5 and D1 receptors in MSN of the direct pathway in response to dopaminergic denervation is shown in box. Within the heteromeric complex, D1 receptors share with mGlu5 receptors the ability to stimulate a PLCβ/ERK pathway underlying L-DOPA-induced dyskinesias (LIDs)^86^. For simplicity mGlu5 receptors are illustrated as monomers.

mGlu1 and mGlu5 receptors are expressed by SNpc dopaminergic neurons, medium spiny GABAergic neurons, cholinergic interneurons, globus pallidus (GP) GABAergic neurons, and subthalamic nucleus (STN) glutamatergic neurons^44^. mGlu1 receptors are expressed at high levels in the SNpc and cerebellum^45^. Stimulation of mGlu1 receptors restrains the activity of nigrostriatal neurons, by activating a Ca^2+^-dependent K^+^ conductance^46^. In striatal projection neurons, mGlu5 receptors are associated with A_2A_ adenosine receptors, and the combined activation of mGlu5, NMDA and A_2A_ receptors counteracts the inhibitory action of D2 receptors on the indirect pathway (Fig. 2).

**Fig. 3:**
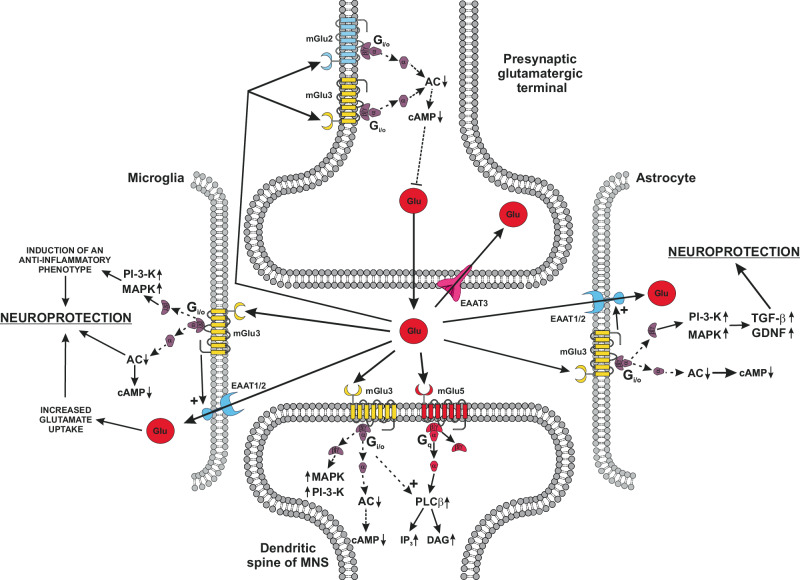
Pleiotropic actions of mGlu3 receptors and potential relevance to the pathophysiology and treatment of PD. Presynaptic mGlu3 receptors are localized in the preterminal region of the axon terminal, and share with mGlu2 receptors the ability to inhibit glutamate release. This mechanism converges with the anti-inflammatory effect in microglia and the induction of TGF-β and GDNF in astrocytes to support the potential disease-modifying effect of mGlu3 receptor agonists or PAMs in PD. Postsynaptic mGlu3 receptors functionally interact with mGlu5 receptors, a mechanism implicated in the potential pro-cognitive effects of mGlu3 receptor agonists or PAMs. For simplicity, mGlu3 receptors are illustrated as monomers.

Group II mGlu receptor subtypes (mGlu2 and mGlu3) are coupled to G_i/o_ proteins, and their activation inhibits adenylyl cyclase activity and negatively modulates voltage-sensitive Ca^2+^ channels. Both receptors are found in the preterminal region of axon terminals, where they inhibit neurotransmitter release^33^ (Fig. 3). Presynaptic mGlu2/3 receptors are not easily accessible to synaptic glutamate, and might be activated by the glutamate released from astrocytes *via* the glutamate:cysteine antiporter^47^. In the basal ganglia motor circuit, presynaptic mGlu2/3 receptors inhibit glutamate release from cortico-striatal fibers, acetylcholine release from striatal cholinergic interneurons, glutamate release at the synapse between excitatory neurons of the STN and the substantia nigra pars reticulata (SNpr)^44,48–50^. The convergence of these effects underlies the symptomatic activity of mGlu2/3 receptor agonists in preclinical models of Parkinsonism^44^.

mGlu3 receptors are also found in post-synaptic densities, and they functionally interact with mGlu5 receptors by boosting mGlu5 receptor signaling (Fig. 3). This mechanism might concur to excitotoxic neuronal damage and generation of LIDs^51–53^. However, the overall effect of mGlu3 receptor activation is neuroprotective because mGlu3 receptors expressed in astrocytes stimulate the production of neurotrophic factors, such as transforming growth factor-β (TGF-β)^54^ and glial-derived neurotrophic factor (GDNF)^55^ (Fig. 3), and activation of mGlu3 receptors drives microglial cells towards an anti-inflammatory phenotype^56,57^ (Fig. 3). mGlu3 receptors display the highest affinity for glutamate among all ionotropic and mGlu receptors, and might respond to ambient glutamate concentrations in the nanomolar range^58^.

**Fig. 4:**
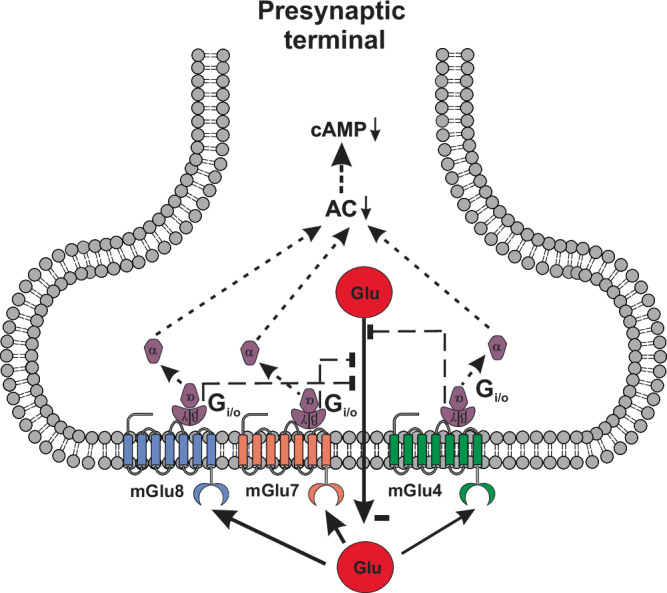
Group III mGlu receptors (mGlu4, mGlu7, and mGlu8 receptors) are found in the active zone of presynaptic terminals, where their activation inhibits neurotransmitter release.

Group III mGlu receptor subtypes (mGlu4, mGlu6, mGlu7, and mGlu8) are all coupled to G_i/o_ proteins and their activation inhibits cAMP formation. mGlu4, mGlu7, and mGlu8 receptors are found in nerve terminals, and their activation inhibits neurotransmitter release (Fig. 4). mGlu4, mGlu7 and mGlu8 receptors show a widespread expression in the basal ganglia motor circuit. These receptors are presynaptically localized in cortico-striatal glutamatergic terminals, and in GABAergic terminals afferent to the internal globus pallidus (iGP), external globus pallidus (eGP) and SNpr, inhibiting both glutamate and GABA release^33,34^. Activation of mGlu7 receptors inhibits GABA release at the striatal:SNpr synapse, thereby restraining the activity of the direct pathway of the basal ganglia motor circuit^59^. However, this regulatory mechanism may only be engaged if extracellular glutamate concentrations are very high because mGlu7 receptors display a very low affinity for glutamate^58,60^.

**Fig. 5:**
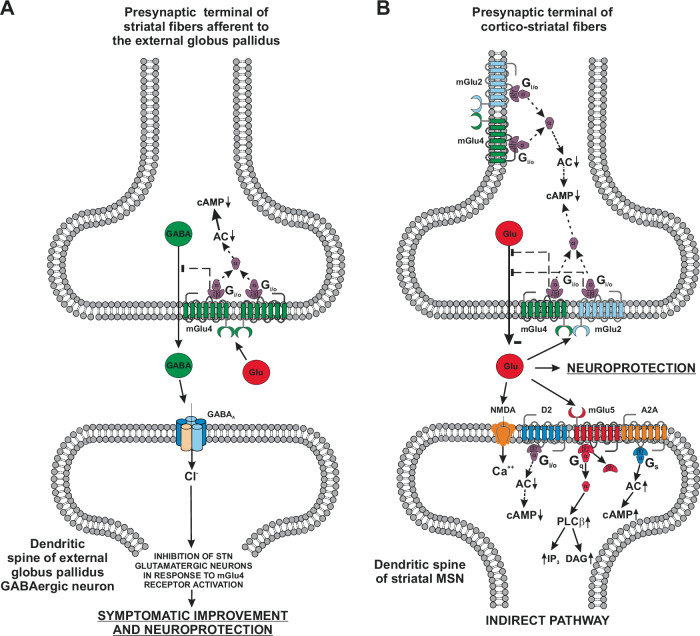
Inhibition of GABA release by homodimeric mGlu4 receptors at the synapse between striatal projection neurons of the indirect pathway and neurons of the external globus pallidus. Through this mechanism, mGlu4 receptor agonists or PAMs may improve motor symptoms in PD and restrain excitotoxic degeneration of nigral neurons (**A**). Inhibition of glutamate release by heterodimeric mGlu2/mGlu4 receptors in cortico-striatal fibers (**B**). Whether mGlu2/mGlu4 heteromers are localized in the pre-terminal region of the axon terminal or in the active zone of neurotransmitter release is unknown.

mGlu4 receptors are expressed presynaptically in the striato-pallidal synapse of the indirect pathway, and their activation inhibits GABA release^61–66^ (Fig. 5A). In the neostriatum, almost 70% of mGlu4 receptor-immunoreactive cortico-striatal terminals target projection neurons of the indirect pathway^67^ (Fig. 5B). The combined inhibition of GABA release in the striato-pallidal synapse and glutamate release in the cortico-striatal synapse restrains the activity of the indirect pathway, accounting for the antiparkinsonian effect of mGlu4 receptor activation^64,66,68,69^ (see below).

## Targeting mGlu5 receptors to restrain neuroinflammation and neurotoxicity in PD

mGlu5 receptors are physically and functionally linked to NMDA receptors, and play a key role in mechanisms of activity-dependent synaptic plasticity underlying learning and memory processes^70,71^. However, an excessive activation of mGlu5 receptors may cause excitotoxic neuronal death^41,72^, although the precise role of mGlu5 receptors in mechanisms of neurodegeneration/neuroprotection is context-dependent^73^. We have shown that endogenous activation of mGlu5 receptors contributes to nigro-striatal degeneration in mice challenged with MPTP^74^. Similar findings were found in primates, where systemic treatment with the mGlu5 receptor negative allosteric modulator (NAM), 3-[(2-methyl-1,3-thiazol-4-yl) ethynyl] pyridine (MTEP), protects dopaminergic neurons of the SNpc, noradrenergic neurons of the locus coeruleus, and the A5/A7 nuclei against MPTP toxicity^75^.

This finding is interesting because depletion of noradrenergic neurons has been implicated in the pathophysiology of non-motor symptoms in PD (see below).

The simplest explanation for these findings is that mGlu5 receptor blockade reduces the excitatory overdrive in the SNpc, and perhaps noradrenergic nuclei, in PD^31,76^. However, mGlu5 receptors are also found in reactive astrocytes and activated microglia, and, in both cell types, receptor expression was found to be upregulated in a neonatal rat model of excitotoxicity characterized by extensive neuroinflammation^43,77^. In astrocytes, activation of mGlu5 receptors downregulates the expression of the glial glutamate transporter, GLAST and GLT-1^78^ (now named excitatory amino acid transporter, EAAT1 and EAAT2, respectively) (Fig. 2), and inhibition of this mechanism might restrain neuronal damage by reducing synaptic glutamate levels. In contrast, microglial mGlu5 receptors appear to reduce neuroinflammation and neurotoxicity (Fig. 2). Accordingly, selective activation of mGlu5 receptors with the orthosteric agonist, (*RS*)-2-chloro-5-hydroxyphenylglycine (CHPG), significantly reduced lipopolysaccharide (LPS)-induced microglial activation and neurotoxicity^79^. In addition, the anti-cancer drug triptolide restrains microglial activation by upregulating mGlu5 receptors in the LPS model of Parkinsonism^80^. Thus, mGlu5 receptor NAMs might enhance microglia-induced neuroinflammation in PD. Of note, there are models of Parkinsonism in which mGlu5 receptor blockade fails to cause neuroprotection. For example, systemic treatment with the mGlu5 receptor NAM, 2-methyl-6-(phenylethynyl)pyridine (MPEP), in rats challenged with 6-hydroxy-dopamine (6-OH-DA) reduced akinesia, without affecting neuronal survival or neuroinflammation^81^.

## Targeting mGlu5 receptors in the treatment of LIDs

LIDs are abnormal involuntary movements, which may occur at the time of maximal plasma concentrations of L-DOPA (peak-dose dyskinesias), or, less frequently, during the ascending and decreasing phases of plasmatic L-DOPA levels (diphasic dyskinesias)^82^. The pathophysiology of LIDs is complex, and likely reflects the formation of a maladaptive form of synaptic plasticity leading to a hyperactivity of striatal projection neurons of the direct pathway expressing both D1 and mGlu5 receptors^83,84^. The seminal work by Paolo Calabresi and his associates has shown that induction of LTP at the synapse between cortico-striatal fibers and striatal projection neurons of the direct pathway is mediated by D1, NMDA, mGlu5 and mGlu1 receptors^85^. An increased glutamatergic tone in the striatum and SNpr has been shown in rodent models of parkinsonian patients with LIDs^86^.

The NMDA receptor antagonist amantadine shows efficacy in reducing LIDs in experimental animals and PD patients, and is the only drug approved for the treatment of LIDs. However, mGlu5 receptor blockade is an interesting option because, as opposed to NMDA receptors, mGlu5 receptors modulate, and not mediate, excitatory synaptic transmission^34^. Interestingly, D1 and mGlu5 receptors form functional heterodimers, which are upregulated in the dopamine denervated striatum. This results into a mechanism of signaling bias, in which D1 receptors stimulate phosphoinositide (PI) hydrolysis instead of enhancing cAMP formation. In turn, this leads to an overactivation of the extracellular signal-related kinase (ERK) pathway with ensuing formation of LIDs^87^ (Fig. 2). Cortical mGlu5 receptors may also be involved in the pathophysiology of LIDs, as suggested by a positron emission tomography (PET) study showing changes in the density of mGlu5 receptors in the motor and somatosensory cortex of “parkinsonian” rats treated with L-DOPA^88^. The use of selective mGlu5 receptor NAMs supports the key role for mGlu5 receptors in the generation of LIDs. For example, in a rat model of LIDs, treatment with MTEP reduces striatal mRNA levels of both prodynorphin and proenkephalin, which are biochemical markers of projection neurons of the direct and indirect pathway, respectively^89^. Treatment with MTEP also reduced LIDs and the associated increase in the activity of the direct pathway, assessed by measuring extracellular GABA levels in the SNpr^90^. mGlu5 receptor expression was found to be increased in the striatum of MPTP-lesioned monkeys developing dyskinesias in response to L-DOPA, as well as in the putamen, iGp and eGP of PD patients with LIDs^91^. mGlu5 receptor NAMs reduced LIDs in MPTP-lesioned monkeys without affecting the therapeutic efficacy of L-DOPA^82–98^.

One of the leading hypotheses on the pathogenesis of LIDs states that under conditions of extensive dopaminergic denervation L-DOPA is taken up by striatal serotonergic fibers, where it is converted to dopamine by LAAD-mediated decarboxylation^99^. Hence, agonists of autoreceptors that inhibit the activity of serotonergic neurons (i.e., 5-HT_1A/B/D_ serotonin receptor subtypes) hold promise for the treatment of LIDs^100,101^.

The association between 5-HT_1A/B_ serotonin receptor PAMs and mGlu5 receptor NAMs might represent a new valuable approach in the treatment of LIDs^102^.

### Clinical studies with mGlu5 receptor NAMs in PD patients with LIDs

Preclinical studies paved the way to the clinical development of two mGlu5 receptor NAMs, mavoglurant (AFQ-056) and dipraglurant, for the treatment of LIDs. Data of 6 randomized clinical trials with mavoglurant vs. placebo have been published^103–106^. All studies enrolled patients with PD and moderate/severe LIDs (items 32 and 33 of UPDRS-IV), and the use of antipsychotic or antidyskinetic drugs was not allowed in the 15 days preceding randomization. However, the duration of treatment ranged from 2 to 13 weeks and the dose of mavoglurant from 20 to 300 mg daily in the different studies. Efficacy measures were (i) patient diaries reporting information on the duration of “off-time” “on-time”, “on-time without dyskinesia”, “on-time with non-troublesome dyskinesia”, and “on-time with troublesome dyskinesia”; (ii) the modified abnormal involuntary movement scale (mAIMS); (iii) UPDRS part 3 and 4 (items 32 and 33); and, (iv) the Lang-Fahn activities of daily living dyskinesia scale (LFALDS). An accurate metanalysis taking into account all the possible bias that might have influence the data was recently published^107^. In the pooled data, treatment with mavoglurant caused no changes in the off-time, on-time, and on-time with troublesome dyskinesia; in one study^106^ mavoglurant was superior to placebo in enhancing the duration of on-time without dyskinesia. Pooled data showed no effect of mavoglurant on the LFADS scale and UPDRS-IV (items 32 and 33). However, mavoglurant caused a small but significant improvement in LIDs evaluated with the mAIMS^107^. Mavoglurant was in general well tolerated, with dizziness, euphoria, fatigue, hallucination, insomnia, and nausea being the most frequent adverse effects. Only changes in dizziness (RR = 4.19) were statistically significant^107^. The metanalysis provided class-one evidence that mavoglurant did not improve LIDs^107^. The clinical development of mavoglurant for the treatment of LIDs was terminated and the drug has been repositioned for the treatment of cocaine use disorder^108,109^.

A phase 2a, double-blind, dose escalation study with dipraglurant was more promising, with the drug reducing peak dose dyskinesias on day 1 and 14 of treatment with an acceptable profile of safety and tolerability^110^ (Table 1). An ongoing study is evaluating the safety and efficacy of a 12-week treatment with dipraglurant in PD patients with dyskinesia (NCT04857359).

### mGlu5 receptor ligands in other dystonic/dyskinetic disorders

Perl et al. ^111^ examined the effect of the mGlu5 receptor NAM, fenobam, and the mGlu5 receptor positive allosteric modulator (PAM), 3-cyano-N-(1,3-diphenyl-1H-pyrazol-5-yl)benzamide (CDPPB), in mutant dt^sz^ hamsters, which develop age-dependent dystonia. Neither drug had any effect on the severity of dystonia, but CDPPB induced axial dyskinesias in mutant hamsters^111^. Thus, at least in this model, mGlu5 receptors appear to be linked to the pathophysiology of dyskinesias, rather than dystonias. Interestingly, genetic deletion of mGlu5 receptors worsens some biochemical and behavioral alterations in BACHD mice, which model Huntington’s disease (HD)^112^, whereas pharmacological activation of mGlu5 receptors with the PAMs, CDPPB or [6,7-dihydro-2-(phenox-ymethyl)oxazolo[5,4-c]pyridin-5(4H)-yl](fluorophenyl)methanone (VU0409551), improved the pathological phenotype of BACHD mice^113,114^. In contrast, a 12-week treatment with the mGlu5 receptor NAM, 2-chloro-4-((2,5-dimethyl-1-(4-(trifluoromethoxy)phenyl)-1H-imidazol-4-yl)ethynyl)pyridine (CTEP), prevented disease progression in the zQ175 mouse model of HD^115^. In a randomized, double-blind, placebo controlled, clinical study, a 32-day treatment with the mGlu5 receptor NAM, mavoglurant, failed to reduce choreatic movements in patients affected by HD^116^. These contrasting findings do not encourage the development of mGlu5 receptor ligands for the treatment of HD.

## The mGlu1 receptor: an unexplored potential drug target in PD

The mGlu1a receptor, the main splice variant of mGlu1 receptors^45^, is expressed in the SNpc and SNpr of rodents and primates^117^, and the mGlu1 receptor transcript is reduced in the rat SNpc after 6-OH-DA-induced nigrostriatal lesions^118^. Longitudinal PET studies in transgenic mice carrying the A53T mutation of human α-synuclein gene showed a transient increase in striatal mGlu1 receptors preceding the decline in motor activity followed by a large decrease in receptor expression. Changes in mGlu1, but not mGlu5, receptor expression showed a high correlation with motor symptoms and the expression of the high affinity dopamine transporter (a marker of integrity of striatal dopaminergic fibers)^119^. Although this evidence suggests that mGlu1 receptors shape the vulnerability of nigro-striatal dopaminergic neurons, pharmacological blockade of mGlu1 receptors failed to protect the nigro-striatal system against MPTP toxicity in mice^74^. Interestingly, neuregulin 1 (NRG1), a trophic factor that regulates neurodevelopment and synaptic plasticity, selectively increases mGlu1 receptor-activity in mesencephalic dopaminergic neurons by inducing the synthesis and membrane trafficking of mGlu1 receptors through the activation of the ErbB4-mediated phosphatidylinositol-3-kinase-Akt-mammalian target of rapamycin (PI-3-K-Akt-mTOR) pathway^120^. This finding links mGlu1 receptors to the biological function of NRG1, known to have a protective role in mouse models of Parkinsonism^121^. Finally, it has been reported that dopamine depletion downregulates mGlu1 receptors in striatal cholinergic interneurons at synapses where dopamine is co-released with glutamate. The loss of mGlu1 receptors in cholinergic interneurons might contribute to motor impairments in early-stage of Parkinsonism in mice^122^. Taken together, these data suggest that mGlu1 receptors might be targeted at least by symptomatic drugs in the treatment of PD, although the precise role of mGlu1 receptors in the regulation of synaptic transmission and plasticity in the basal ganglia motor circuit remains to be elucidated. This warrants further studies with potent and selective mGlu1 receptor PAMs or NAMs such as compounds N-(4-(trifluoromethyl)oxazol-2-yl)-9H-xanthene-9-carboxamide (Ro 07-11401), 3-chloro-N-[3-chloro-4-(4-chloro-1,3-dihydro-1,3-dioxo-2H-isoindol-2-yl)phenyl]-2-pyridinecarboxamide (VU0483605), and N-(3-chloro-4-(4-chloro-1,3-dioxoisoindolin-2-yl)phenyl)-3-methylpicolinamide (VU0483737), and 2-cyclopropyl-5-[1-(2-fluoro-3-pyridinyl)-5-methyl-1H-1,2,3-triazol-4-yl]-2,3-dihydro-1H-isoindol-1-one (CFMTI) and (3,4-dihydro-2H-pyrano[2,3-b]quinolin-7-yl)(cis-4-methoxycyclohexyl)-methanone (JNJ16259685), respectively^123^.

## Activation of mGlu3, but not mGlu2, receptors restrains neuroinflammation and neurotoxicity in PD

Early studies of mGlu2 and mGlu3 receptors on mechanisms of neurodegeneration/neuroprotection in the basal ganglia motor circuit have been confounded by the use of orthosteric agonists, such as (1R,4R,5S,6R)-4-amino-2-oxabicyclo[3.1.0]hexane-4,6-dicarboxylic acid (LY379268), that does not differentiate between the two receptor subtypes^60^. For example, LY379268 has been used for the study of long-term depression (LTD) of excitatory synaptic transmission at the synapse between the STN and SNpr. The use of mGlu2 and mGlu3 receptor knockout mice allowed to establish that this form of synaptic plasticity was mediated by mGlu2 receptors^124^. Treatment with LY379268 was neuroprotective in the 6-OH-DA and MPTP models of Parkinsonism^125,126^, and also reduced akinesia in reserpine-treated rats^125^. What the two receptors have in common is their ability to inhibit neurotransmitter release from nerve terminals. However, mGlu2 and mGlu3 receptors diverge in the regulation of glial-mediated mechanisms that shape neuroinflammation and toxicity. Only mGlu3 receptors are expressed by astrocytes, and their activation enhances the production of TGF-β and GDNF (Fig. 3). Through this mechanism, activation of mGlu3 receptors in cultured astrocytes protects neurons against excitotoxic death^32,54^. In contrast, selective activation of mGlu2 receptors was shown to be harmful to neurons in in vitro models and in mice challenged with MPTP^127,128^. GDNF is a potent neurotrophic factor for survival and axonal growth of mesencephalic dopaminergic neurons, and has powerful neuroprotective and neurorestorative properties because it promotes recovery of the lesioned nigro-striatal system and improves motor functions in both rodents and non-human primates^129–131^. The neuroprotective action of GDNF is likely mediated by TGF-β, suggesting that activation of mGlu3 receptors in astrocytes promotes a powerful mechanism of neuroprotection mediated by two synergic neurotrophic factors^55,132^. The potential clinical relevance of this mechanism is supported by the development of strategies for the CNS delivery of GDNF in the treatment of PD. A phase 1 study showed that surgical delivery of adeno-associated virus (AAV2) encoding GDNF into the putamen of subjects with advanced PD was safe and well tolerated (NCT01621581). A phase 2 study is underway to evaluate the efficacy, safety and biodistribution of AAV2-GDNF in patients with PD (NCT06285643).

mGlu3 receptors are also expressed by microglia, and their activation drives microglia differentiation towards an anti-inflammatory phenotype (Fig. 3). Perinatal brain injury induced by gestational low-protein diet (LPD) combined with interleukin-1β injection (IL-1β) in rats caused a substantial downregulation of mGlu3 receptors in microglia resulting into neuroinflammation. Genetic deletion or pharmacological blockade of mGlu3 receptors in microglia mimicked the proinflammatory phenotype induced by LPD/IL-1β^56^. Microglial mGlu3 receptors may also protect against neuroinflammation associated with PD, as suggested by the evidence that mice lacking mGlu3 receptors were more sensitive to MPTP-induced nigrostriatal damage, and showed an increased microglial activation in the SNpc and a reduced expression of genes encoding anti-inflammatory cytokines in the striatum^57^. We found that two haplotypes of GRM3 (the gene encoding the mGlu3 receptor) were associated with PD, and that individual gene variants correlated with motor and non-motor signs of PD. In addition, patients carrying one of the PD-associated haplotypes showed an impaired cortical plasticity evaluated by magnetic transcranial stimulation^57^. This strongly encourages the development of mGlu3 receptor-selective PAMs as disease-modifying agents in PD. These drugs are expected to restrain neuroinflammation, and halt the progression of nigrostriatal degeneration in experimental models of Parkinsonism.

## Targeting group III mGlu receptors for the treatment of motor symptoms in PD

### Preclinical studies with mGlu4 receptor ligands in models of Parkinsonism

A large body of evidence indicates that activation of mGlu4 receptors provides a valuable strategy to improve motor symptoms in PD by restraining the activity of the indirect pathway of the basal ganglia motor circuit (see above).

Systemic treatment with *N*-phenyl-7-(hydroxyimino)cyclopropa[b]chromen-1a-carboxamide (PHCCC), N-(3-chlorophenyl)picolinamide (VU0364770) or 5-methyl-N-(4-methylpyrimidin-2-yl)-4-(1H-pyrazol-4-yl)thiazol-2-amine (ADX88178), which are all selective mGlu4 receptor PAMs, was found to reverse reserpine-induced akinesia^65^, haloperidol-induced catalepsy, and forelimb asymmetry in 6-OH-DA lesioned rats^133,134^. The mGlu4 receptor PAM, ((1S,2R)-N1-(3,4-dichlorophenyl)cyclohexane-1,2-dicarboxamide) (Lu AF21934), inhibited cortico-striatal synaptic transmission, reduced haloperidol-induced catalepsy, and lowered the threshold of L-DOPA in improving akinesia^133,135^. Interestingly, mGlu4 receptors can form heteromeric complexes with mGlu2 receptors expressed in the cortico-striatal pathway, whereas homodimeric mGlu4 receptors predominate at the striato-pallidal synapses^136–139^ (Fig. 5A,B). Striato-pallidal mGlu4 receptors should be targeted in the treatment of PD because a robust antiparkinsonian effect was reported with compound N-(3-chloro-4-fluorophenyl)-1H-pyrazolo[4,3-b]pyridin-3-amine (VU0418506), which selectively amplifies the activity of mGlu4 homodimers^136^. Orthosteric group III mGlu receptor agonists, such as (2S)-2-amino-4-{hydroxy[hydroxy(4-hydroxy-3-methoxy-5-nitrophenyl)methyl] phosphoryl} butanoic acid (LSP1-2111), have also been proposed as potentially useful agents in the treatment of PD. LSP1-2111 displays a preferential affinity for mGlu4 receptors, it was found to restrain striato-pallidal transmission and its systemic administration reversed haloperidol-induced catalepsy^63^. The interest in mGlu4 receptor activation is growing more and more, and a novel radiolabeled ligand, [^11^C]PXT012253, has been developed for the study of mGlu4 receptors in the monkey brain. The use of this PET tracer in human studies is warranted^140^.

The ability of mGlu4 receptors to negatively modulate glutamate release from cortico-striatal fibers^134^ suggests a potential use of mGlu4 receptor PAMs or orthosteric agonists in the treatment of LIDs (see above). However, neither the mGlu4 receptor PAM, VU0364770, nor the mGlu4 receptor orthosteric agonist, LSP1-2111, showed antidyskinetic activity in rats lesioned with 6-OH-DA and treated with L-DOPA, and only VU0364770 enhanced the therapeutic efficacy of L-DOPA^141^. Perhaps the combined use of a selective mGlu4 receptor PAM may have an indirect beneficial effect on LIDs allowing to reduce the daily dose of L-DOPA in PD patients.

### The mGlu4 receptor PAM, foliglurax, failed to improve motor symptoms in PD patients

The highly potent and selective mGlu4 receptor PAM, PXT002331/foliglurax, showed a high brain exposure after oral administration^142^, and improved motor symptoms and LIDs in macaques challenged with MPTP^143^. Foliglurax is fully active at mGlu4 homodimers, but fails to enhance responses at mGlu2/mGlu4 and mGlu3/mGlu4 heterodimers^144^. So far, foliglurax has been the only mGlu4 receptor ligand clinically developed for the treatment of PD. In a phase 2a double-blind, randomized study, 153 patients with advanced PD (disease duration >3 years) treated with a stable dose of L-DOPA were randomly assigned to receive either 10 or 30 mg foliglurax or placebo twice a day for 28 days. The primary endpoint was the change in off-time assessed by the Hauser diaries. Secondary endpoints were changes in the UPDRS and the on-time without troublesome dyskinesia. Foliglurax treatment did not cause significant changes in the off-time, and secondary endpoints were also not met, and the development of foliglurax for the treatment of PD was terminated^145,146^. However, in the phase 2a study, the least-squares differences in daily awake time were greater with foliglurax vs. placebo, and 44.7% of patients treated with 30 mg foliglurax had a decrease in daily awake off-time >1 h vs. 30.4% with placebo^146^. Perhaps the termination of the foliglurax program has been premature.

### Targeting mGlu4 receptors to restrain neurotoxicity and neuroinflammation

mGlu4 receptor activation reduces GABA release in striato-pallidal synapses, an effect that may restrain the overactivation of STN neurons, and, therefore, excitotoxic neuronal death in the SNpc (see above). Using the MPTP model in mice, we could demonstrate a neuroprotective activity of the mGlu4 receptor PAM, PHCCC^147^. Interestingly, PHCCC could also induce neuroprotection when locally infused in the eGP^147^. Neuroprotection could also be observed in response to systemic treatment with 3 mg/kg of foliglurax in mice challenged with MPTP^148^. In 6-OH-DA-lesioned mice, pharmacological activation of mGlu4 receptors could also induce neuroprotection associated with an anti-inflammatory response^149,150^.

mGlu4 receptor activation may have a profound impact on mechanisms of innate and adaptive immunity underlying neuroinflammation in PD. In mice developing experimental autoimmune encephalomyelitis (EAE, a model of multiple sclerosis), genetic deletion of mGlu4 receptors enhanced EAE severity and associated neuroinflammation in the spinal cord, whereas treatment with PHCCC in wild-type mice was protective^151^. Neuroprotection was also observed in EAE mice treated with the mGlu4 receptor PAM, ADX88178^152^. It has been demonstrated that activation of mGlu4 receptors in antigen-presenting cells drives T cell differentiation into T regulatory cells underlying immune tolerance^151^. Interestingly, application of ADX88178 to cultured microglial cells could also attenuate microglial activation and inflammation in response to LPS^153^. Thus, drugs that selectively activate mGlu4 receptors cater the potential to behave as disease-modifying agents in PD by reducing neuroinflammation and excitotoxic neuronal death.

### Study of mGlu7 and mGlu8 receptors in preclinical models of Parkinsonism

The mGlu7 receptor PAM, N,N’-bis(diphenylmethyl)-1,2-ethanediamine dihydrochloride (AMN082), showed anti-Parkinsonian activity in reversing reserpine- and haloperidol-induced akinesia^154–156^. However, in some studies, AMN082 produced electrophysiological and behavioral effects in mice lacking mGlu7 receptors, suggesting that the drug may have off-target effects^157^. The mGlu8 receptor orthosteric agonist, (S)-3,4-dicarboxyphenylglycine (DCPG), failed to reduce catalepsy and akinesia induced by an acute treatment with haloperidol or reserpine, but became highly effective in animals repeatedly challenged with haloperidol or reserpine^154,158^. These findings suggest that adaptive changes occurring in the basal ganglia motor system in response to dopamine depletion or dopamine receptor blockade shape the behavioral response to mGlu8 receptor activation. In addition, mGlu8 receptors display high affinity for glutamate^58^, which can compete with DCPG in behavioral studies.

In conclusion, the study of mGlu7 and mGlu8 receptors in the pathophysiology and treatment of PD requires further investigation with the aid of more selective and appropriate pharmacological tools.

## mGlu receptors as a potential target in the treatment of non-motor symptoms of PD

PD is associated with a broad-spectrum of non-motor symptoms (NMS). Some NMS, such as hyposmia, rapid eye movement sleep behavior disorder, excessive daytime sleeping, depression and constipation may precede motor symptoms by several years. Other NMS, such as global cognitive impairment, alterations in executive function and working memory, autonomic dysfunction and sleep disorders are more frequent in late stages of PD^159^. Hallucinations, dysphagia, and cognitive impairment develop as a result of the complex interactions between progressive neuronal degeneration and dopaminergic replacement therapy. Sensory symptoms, like pain and hyposmia, are more common in early-onset PD, whereas depression and anxiety are seen in all stages of the disease and are linked to the OFF phase in patients with motor fluctuations. Autonomic dysfunction including bladder, bowel and sexual dysfunction, and cardiovascular dysautonomia, usually precedes motor symptoms, but become more relevant with disease progression^160^. Targeting mGlu receptors may offer a new potential strategy for the management of NMS in PD. Cognitive impairment in PD has been linked to functional abnormalities in glutamatergic transmission and plasticity^161^, and might reflect alterations in the signal-to-noise ratio in learning and memory processes. This may help explain the paradoxical improvement of cognition observed with drugs acting as fast NMDA receptor channel blockers, such as memantine and amantadine. Memantine is approved worldwide for the treatment of Alzheimer’s disease, and improves attention and episodic memory in disorders characterized by dementia and Parkinsonian features, such as Lewy body dementia and Parkinson’s Disease Dementia^162,163^. Treatment with amantadine could enhance the speed of visual cognitive processing in non-Caucasian PD patients^164^. mGlu5 receptors are physically and functionally linked to NMDA receptors and cooperate with NMDA receptors in the regulation of activity-dependent synaptic plasticity^165^. Hence, we can predict that mGlu5 receptors behave similarly to NMDA receptor antagonists in improving cognition in chronic neurodegenerative disorders. Pharmacological blockade or genetic deletion of mGlu5 receptors was found to improve cognitive function in mouse models of Alzheimer’s disease^166,167^. However, this effect might be an indirect consequence of neuroprotection because oligomeric forms of the β-amyloid peptide (Aβ_1-42_) cause neurotoxicity by interacting with mGlu5 receptors and the cellular prion protein in neuronal membranes^165–167^. In rats locally injected with MPTP in the SNpc, treatment with the mGlu5 NAM, MPEP, improved cognition in the T maze and in an object recognition test, and was also protective against nigrostriatal damage, neuroinflammation, and loss of CA1 hippocampal neurons^168^. Studies in which a pro-cognitive effect is dissociated from neuroprotection are needed to disclose the activity of mGlu5 receptor antagonists on cognitive function in models of Parkinsonism.

The mGlu3 receptor is another potential drug target to improve cognition in PD. Postsynaptic mGlu3 receptors interact with mGlu5 receptors by boosting mGlu5 receptor-mediated PI hydrolysis^51^. This interaction is involved in the induction of LTD in the prefrontal cortex (PFC)^51,169^, a form of activity-dependent synaptic plasticity that underlies PFC-dependent cognitive functions^169–171^. Genetic variants of GRM3 are associated with lower performance in PFC-dependent cognitive tasks^171–173^.

Interestingly, activation of mGlu3 receptors with the endogenous agonist, N-acetylaspartylglutamate (NAAG), enhances delay in cell firing in the dorsolateral PFC during a working memory task, improving cognitive function^174^. In virally-suppressed HIV-infected individuals with persistent cognitive impairment, higher NAAG levels in the frontal white matter were associated with better attention and working memory, and higher NAAG levels in the left basal ganglia were related to a better verbal fluency^175^. Interestingly, we found an association between an intronic polymorphic variant of GRM3 (rs1989796, C>T) and attention and memory in patients with PD^57^. Taken together, these findings encourage the development of selective mGlu3 receptor agonists or PAMs for the treatment of cognitive dysfunction in PD.

Chronic pain is another NMS, which has a high impact on the quality of life, occurs in >80% of PD patients, and is difficult to treat. The King’s PD Pain Scale recognizes 7 pain domains: musculoskeletal, chronic, fluctuation-related, nocturnal, orofacial, discoloration (edema/swelling) and radicular pain, but the exact pathophysiological mechanisms underlying each of these domains are unknown^176,177^. Glutamate has a key role in pain transmission and in the induction and maintenance of nociceptive sensitization underlying chronic pain^171,178^. Multiple mGlu receptor subtypes are involved in the pathophysiology of chronic pain and are potential drug targets for pain management. mGlu5 receptor NAMs are candidate drugs in the treatment of pain associated with PD, because these agents have consistently shown analgesic activity in models of chronic inflammatory and neuropathic pain^179,180^. mGlu1 receptor antagonists also cause analgesia in models of inflammatory and neuropathic pain, but their use was associated with motor impairment and cognitive adverse effects^181–183^. mGlu5 receptor antagonists are more effective than mGlu1 receptor antagonists in reducing anxiety, which is frequently associated with chronic pain^184^.

Drugs that activate mGlu2/3^185,186^, mGlu4^54,187,188^ or mGlu7^189^ receptors have shown analgesic activity in models of chronic inflammatory and/or neuropathic pain. Interestingly, L-acetylcarnitine (LAC), a drug that epigenetically enhances mGlu2 receptor expression, causes long-lasting analgesia in rodents^190,191^, and is used for the treatment of diabetic neuropathy and other painful peripheral neuropathies. This drug has an excellent safety profile and might be repositioned for the treatment of pain associated with PD.

The prevalence of depression exceeds 35% in PD, is associated with freezing of gait, apathy, and fatigue, and frequently occurs in patients with β-glucocerebrosidase mutations^192,193^. Depression in PD reflects a dysfunction of central monoaminergic systems^194^, and, in some patients, can be efficiently managed with a dopaminergic replacement therapy. Selective Serotonin Reuptake Inhibitors (SSRIs) or Serotonin Noradrenaline Reuptake Inhibitors (SNRIs) are drugs of choice in the treatment of depression in PD, but their use may worsen motor symptoms^195^ and enhance apathy^196^. Glutamate receptors have emerged as novel targets for the treatment of depression, and three NMDA receptor antagonists are either approved (S-ketamine, dextromethorphane) or under development (S-methadone) for the treatment of depression^197^. mGlu5 receptor blockade was shown to improve depressive symptoms in animal models^198–200^, and changes in brain density of mGlu5 receptors have been consistently detected by PET imaging or biochemical analysis of post-mortem tissues in individual affected by major depressive disorders^200–205^. The mGlu5 receptor NAM, basimglurant, has been clinically developed for the treatment of major depressive disorder. In a phase 2b, double-blind, randomized clinical trial, a 6-week add-on treatment with basimglurant failed to meet the primary endpoint (change on the score from baseline in the Montgomery-Asberg Depression Rating Scale) in patients with major depressive disorder. However, basimglurant treatment caused significant changes in patient-rated, depression-related secondary endpoints^206^, suggesting that the use of mGlu5 receptor NAMs in depression may be promising and warrants further investigation.

mGlu2 and mGlu3 receptors are also considered as candidate drug targets in the treatment of depression, but data with mGlu2/3 receptor ligands are not uniform. mGlu2/3 receptor antagonists show fast and sustained antidepressant-like effects similar to those of ketamine in rodents^52^, but systemic injection of the mGlu2/3 receptor agonist, LY379268, facilitates the induction of neuroadaptive mechanisms in response to antidepressants^207–209^. In addition, the selective mGlu2 receptor PAM, N-(4-((2-(trifluoromethyl)-3-hydroxy-4-(isobutyryl)phenoxy)methyl)benzyl)-1-methyl-1H-imidazole-4 carboxamide (THIIC), showed anxiolytic/antidepressant efficacy in different assays, indicating that selective activation of mGlu2 receptors may have therapeutic potential by normalizing excessive glutamatergic neurotransmission^210^. LAC, which induces the expression of mGlu2 receptors in the CNS, has consistently shown antidepressant-like activity in animal models of stress-related disorders, and plasma LAC levels are reduced in individual affected by major depressive disorder^211–213^. Perhaps modulation of mGlu2 receptor expression by LAC may provide a new strategy for the combined management of pain and depression in PD.

## Conclusion

**Fig. 6:**
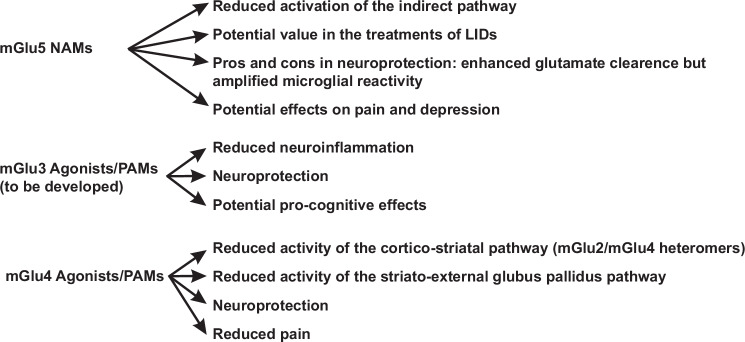
Putative candidate drugs for broad-spectrum treatments of Parkinson’s disease.

Many decades have passed since mGlu receptor identification, but much information is still missing. Modulation of neurotransmission and synaptic plasticity by mGlu receptors is still a potential target for the development of neuroprotective, anti-inflammatory and symptomatic drugs in PD, because these receptors are highly expressed in the basal ganglia motor circuit, and have been implicated in the pathophysiology of motor symptoms, NMS and LIDs (Fig. 6). The mGlu5 receptor NAMs, mavoglurant and dipraglurant, and the mGlu4 receptor PAM, foliglurax, have been clinically developed with disappointing results^104,105,110,145,146^ in spite of promising preclinical studies. A potential explanation may reside in the design of clinical studies, the differential affinity and efficacy of allosteric modulators for mGlu receptor homodimers and heterodimers^214^, or potential off-target effects of mGlu receptor ligands. We believe that mGlu5 receptor NAMs or mGlu4 receptor PAMs still cater the potential to behave as broad-spectrum antiparkinsonian agents, combining symptomatic and disease-modifying effects, taking into consideration that studies of neuroprotection in PD await the identification of early biomarkers of the disease, which allow treatments in the presymptomatic stage. The so called Silent Allosteric Modulators (SAMs) of mGlu5 receptors, which inhibit pathological mGlu5 receptors signaling sparing the physiological actions of mGlu5 receptors (e.g., their role in learning and memory processes)^215,216^ may be even better candidate drugs for PD. The study of mGlu3 receptors in PD is limited by the lack of potent and selective PAMs. These drugs might combine a robust neuroprotective and anti-inflammatory effect with a potential pro-cognitive effect, and their development is supported by the association of GRM3 gene variants and motor and non-motor symptoms in PD patients.

## Data Availability

No datasets were generated or analysed during the current study.
